# Pneumonia-induced endothelial amyloids reduce dendritic spine density in brain neurons

**DOI:** 10.1038/s41598-020-66321-1

**Published:** 2020-06-09

**Authors:** Allison M. Scott, Alexandrea C. Jager, Meredith Gwin, Sarah Voth, Ron Balczon, Troy Stevens, Mike T. Lin

**Affiliations:** 1 Departments of Physiology and Cell Biology, Mobile, Alabama 36688 USA; 2 Biochemistry and Molecular Biology, Mobile, Alabama 36688 USA; 3 Internal Medicine, Mobile, Alabama 36688 USA; 4 Center for Lung Biology, College of Medicine, University of South Alabama, Mobile, Alabama 36688 USA

**Keywords:** Respiration, Infection, Cellular neuroscience

## Abstract

*Pseudomonas aeruginosa* pneumonia elicits endothelial cell release of cytotoxic amyloids that can be recovered from the bronchoalveolar lavage and cerebrospinal fluids of critically ill patients. Introduction of these cytotoxic amyloids into the lateral ventricle impairs learning and memory in mice. However, it is unclear whether the amyloids of lung origin (1) are neurotropic, and (2) cause structural remodeling of hippocampal dendrites. Thus, we used electrophysiological studies in brain slices and structural analysis of post-mortem tissues obtained from animals exposed to endothelium-derived amyloids to assess these issues. The amyloids were administered via three different routes, by intracerebroventricular, intratracheal, and intraperitoneal injections. Synaptic long-term potentiation was abolished following intracerebroventricular amyloid injection. Fluorescence dialysis or Golgi-impregnation labeling showed reduced dendritic spine density and destabilized spines of hippocampal pyramidal neurons 4 weeks after intracerebroventricular amyloid injection. In comparison, endothelial amyloids introduced to the airway caused the most prominent dendritic spine density reduction, yet intraperitoneal injection of these amyloids did not affect spine density. Our findings indicate that infection-elicited lung endothelial amyloids are neurotropic and reduce neuronal dendritic spine density *in vivo*. Amyloids applied into the trachea may either be disseminated through the circulation and cross the blood-brain barrier to access the brain, initiate feed-forward amyloid transmissibility among cells of the blood-brain barrier or access the brain in other ways. Nevertheless, lung-derived amyloids suppress hippocampal signaling and cause injury to neuronal structure.

## Introduction

Many critically ill patients suffer from long-term cognitive impairment that may be associated with the occurrence of delirium during their intensive care unit stay^1,2^. While causative links between delirium and cognitive decline in critically ill patients remain unclear, they cannot be fully ascribed to age, gender, relative brain hypoxia, anesthetics and sedatives, and genetic predispositions^3–7^. However, infection has emerged as an important cause of cognitive impairment, as both community- and hospital acquired pneumonias lead to neurological dysfunction^8,9^. While how learning and memory is impaired by community-acquired pneumonia remains unclear, some progress toward understanding the causes of neurological dysfunction in nosocomial pneumonia has been made. Sepsis leads to shedding of endothelial heparan sulfate from the endothelial glycocalyx, which may then access the brain and impair brain-derived neurotrophic factor-mediated neurological processing^10^. In addition, lung infection leads to generation of amyloid species within the lung itself, and amyloid species can be recovered in the cerebrospinal fluid of infected patients, where they are neurotoxic^11–14^. Heparan sulfate and cytotoxic amyloids may contribute to neurotoxicity separate from and in combination with other causes of neuroinflammation.

We recently quantified amyloids in specimens collected from critically ill patients with and without monomicrobial bacterial pneumonia. Patients diagnosed with nosocomial pneumonia have increased levels of cytotoxic amyloids in their bronchoalveolar lavage fluid and cerebrospinal fluid when compared to noninfected patients^8,14^. As an innate response to bacterial pneumonia infection, pulmonary endothelium produces and releases amyloids^13,14^. Rodents that either received intratracheal *P. aeruginosa* instillation, or intracerebroventricular injection of endothelial amyloids in the absence of bacteria, showed impaired learning and memory^15^. Significantly, whereas the amyloids generated from endothelium exposed to PA103 (a *P. aeruginosa* strain with a functional type 3 secretion system) impair learning, amyloids from endothelium exposed to ΔPcrV (a defective type 3 secretion system *P. aeruginosa* strain) do not. These results suggest that the *P. aeruginosa* type 3 secretion system and effector exoenzymes are required for pulmonary endothelial production and release of cytotoxic amyloids^11–14^. Notably, these amyloids share features of prions, as they are heat stable, RNase and DNase insensitive, protease resistant, transmissible, and self-replicating, and they also show immunoreactivity to the beta-amyloid (Aβ) and tau (τ) antibodies used to detect neuropathologic proteins in Alzheimer’s disease brains^11–15^. Endothelial amyloid neurotoxicity is removable by immunodepleting Aβ and τ complexes using selective antibodies^8,14^. Taken together, these studies suggest that pneumonia-induced cytotoxic amyloids are released from lung, circulate in the blood, and they are present in the cerebrospinal fluid where they might impair brain function well after the primary infection has cleared.

Recent studies have demonstrated that amyloid species purified from diseased brains and injected directly into either the peritoneal cavity or systemic circulation are transmissible and neurotropic^16–18^. More specifically, these amyloids translocate from the circulation to susceptible brain regions and induce amyloid aggregation patterns that are similar to what was seen in the brains they were purified from^19^. However, evidence for how an illness elicits production of peripheral cytotoxic amyloids, i.e., not originating in the brain, that biodistribute through the circulation and access the brain is presently lacking.

In this study, we examined whether infection-elicited cytotoxic amyloids can access the brain following their introduction into the cerebrospinal fluid, trachea, and intraperitoneal space, and further, whether they cause structural remodeling of dendritic spines in the hippocampus. It is well-documented that an anatomical maturation of neuronal dendritic spines and an increase in spine density directly correlate with synaptic plasticity and learning, whereas aging and neuropathologies cause spine pruning^20–24^. Thus, after obtaining the endothelial amyloids post PA103 or ΔPcrV infection *in vitro*, we injected them into the intracerebroventricular (ICV), intratracheal (INT), or intraperitoneal (IP) spaces in mice, recorded the hippocampal field potential, and compared dendritic spine types and their density over 4 weeks.

## Results

### Dendritic spine quantification

**Figure 1:**
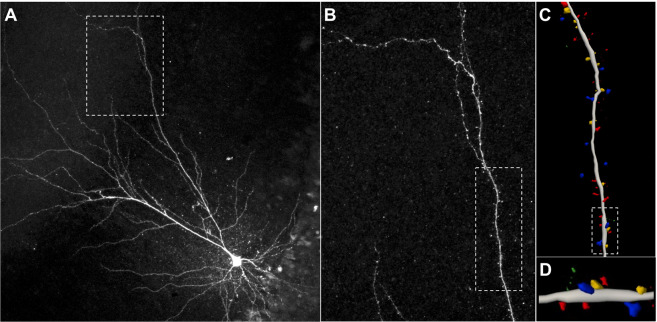
Quantification of dendritic spines. (**A**) Representative photograph of a single section containing a representative Lucifer yellow-filled CA1 pyramidal cell. This image was captured with a 20X objective, and the fluorescence signal in Z-stack projection was averaged. The marquee area is shown in (**B**). (**B**) Amplified sections were captured with a 60X objective and the fluorescence signal was averaged to visualize dendrites and dendritic spines. The marquee area is shown in (**C**). (**C**) 3D reconstruction of the dendrite and spines. (**D**) Enlarged section as indicated in (**C**) shows 4 types of spines classified by their shapes. From most to least mature: mushroom (blue), stubby (yellow), thin (red), and filopodial (green).

To quantify postsynaptic spines on hippocampal CA1 pyramidal neurons, Lucifer yellow, Alexa dye, or biocytin was dialyzed into cells and then analyzed. The results were comparable, so we used Lucifer yellow throughout this study because it was the least likely to precipitate out of solution and plug the patch pipette tips. After cell dialysis, brain slices were fixed and imaged using a confocal laser scanning microscope (Fig. 1A,B). Dendrites and dendritic branches were semi-manually traced (Fig. 1C), and dendritic spines were detected and classified into four spine types—mushroom, stubby, thin, and filopodia (Fig. 1D)—using a custom defined setting described in the Methods.

**Figure 2:**
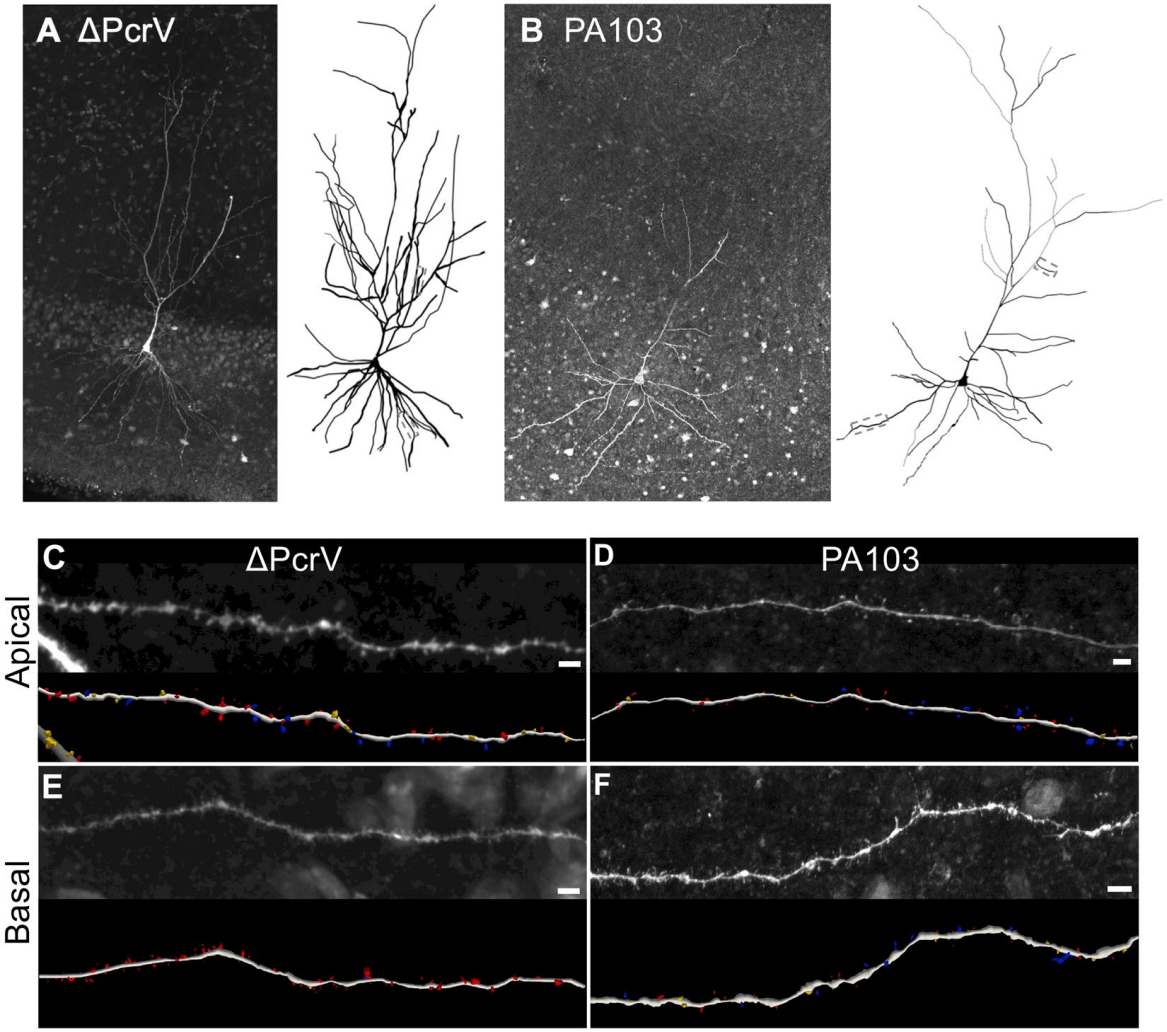
Injection of PA103-amyloid into the cerebral ventricle reduces apical spines. (**A,B**) Photographs of single z-stack sections containing representative Lucifer yellow-filled CA1 pyramidal cells from a ΔPcrV-amyloid (**A**) and a PA103-amyloid (**B**) animal. Images were captured with a 20X objective and show averaged z-stack fluorescence signals. Tracings of the cell body and complete dendritic tree reconstructed from all sections containing the same cells are shown on the right. Marquee areas of an apical and a basal dendrite were captured with a 60X objective and shown in (**C**–**F**). (**C**–**F**) Enlarged micrographs and 3D reconstructions of the apical (**C,D**) or basal (**E**–**F**) dendrites and spines from ΔPcrV-amyloid (**C,E**) or PA103-amyloid (**D,F**) mouse. Types of spines are color-coded: mushroom, blue; stubby, yellow; and thin, red. White horizontal bars indicate 4 µm.

**Figure 3:**
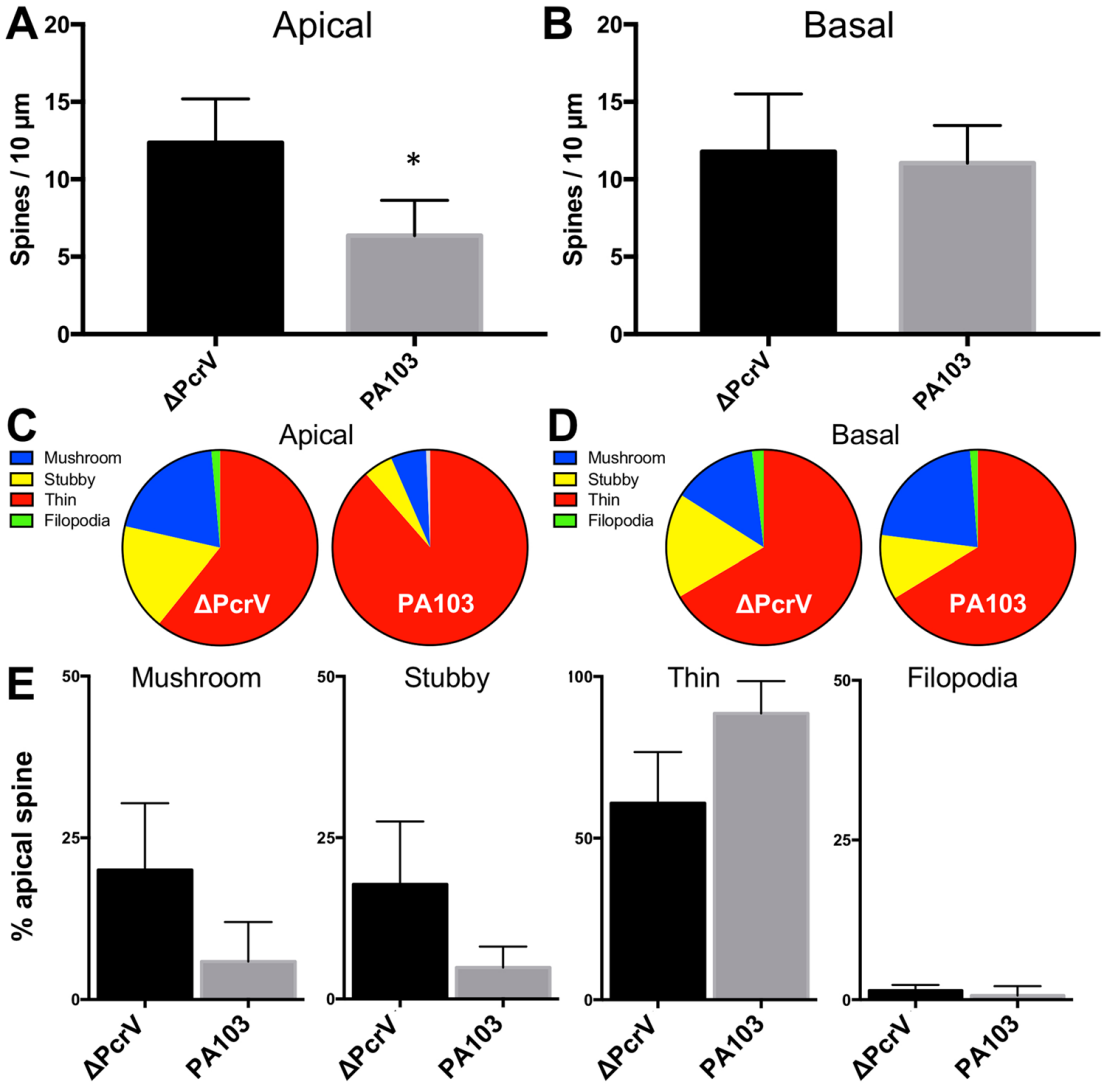
PA103-amyloid in the cerebral ventricle reduces apical spine density and destabilizes apical spines. (**A,B**) Bar graphs depict the mean differences in spine density in the apical (**A**) or basal (**B**) dendritic trees of CA1 pyramidal cells from ΔPcrV- or PA103-amyloid treated mice. (**C,D**) Pie charts show the distribution of spine types in apical (**C**) or basal (**D**) dendrites. (**E**) Bar graphs depict the mean differences in spine types in apical dendrites after animals were treated with ΔPcrV- or PA103-amyloid. *p = 0.006; *t*-test.

To study how endothelial-derived amyloids affect synaptic plasticity and modulate neuronal dendritic spines, we intracerebroventricular (ICV) injected either ΔPcrV- or PA103-amyloid (please see Methods for detailed description and amyloid preparation). Note that ΔPcrV-amyloid or PA103-amyloid represents bacteria-free pulmonary endothelial supernatant collected after the cells were exposed to the respective bacteria. We obtained 6 mouse brains two weeks after ICV injection (3 ΔPcrV-amyloid and 3 PA103-amyloid). Brain slices were acutely prepared for fluorescence dye dialysis and plasticity recordings. We patch-filled 8–12 CA1 pyramidal cells from each brain for more than 10 min while monitoring cell and recording properties every 3 min to ensure continuous dialysis (Fig. 2). Electrophysiological properties showed no obvious differences among neurons of different dendritic tree morphology (e.g., bifurcated vs. non-bifurcated primary dendrite) or neurons located in different depth or portions of the CA1 cell layer. Thus, all neurons with a resting membrane potential below −55 mV that did not have significant drift in resistance properties were included—a total of 23 ΔPcrV-amyloid treated neurons and 20 PA103-amyloid treated neurons were studied. Of these, 9 ΔPcrV-amyloid treated neurons (40%) and 5 PA103-amyloid treated neurons (25%) were determined to be sufficiently filled with Lucifer yellow for analysis of dendritic spine density and type. After patch fill, brain slices were fixed and immunostained for presynaptic terminals. However, immunostaining did not yield clean results, and we were unable to quantify functional synapses (a presynaptic terminal closely localized with a postsynaptic spine). Nevertheless, we were able to trace dendritic trees (Figs. 2A,B) and 3D reconstruct postsynaptic spines in the apical oblique dendrite and basal dendrites for analysis (Fig. 2C–F).

Lucifer yellow-filled, 3D reconstructed, dendritic spine density and type were subsequently quantified. The difference in mean spine density between ΔPcrV- and PA103-amyloid treated neurons was statistically significant on the apical oblique dendrites (12.4 ± 1.3 and 6.4 ± 1.0, respectively; n = 5 cells each, *p* = 0.006, *t*-test; Fig. 3A), but not basal dendrites (11.8 ± 1.7 and 11.0 ± 1.0, respectively; n = 5 cells each, *p* > 0.05, *t*-test; Fig. 3B). The spine types on the apical dendrites also appeared to differ between ΔPcrV- and PA103-amyloid treated neurons, but not on basal dendrites (Fig. 3C,D). Thus, we further compared the apical dendritic spine types, ranging from the most mature mushroom spines, to stubby and thin spines, and to the immature filopodia spines. Figure 3E shows that the spine type distribution shifted from mushroom and stubby spines to thin spines in PA103-amyloid treated neurons, suggesting that PA103-amyloid in the brain destabilizes postsynaptic spines.

### Association of dendritic spine density and long-term potentiation

**Figure 4:**
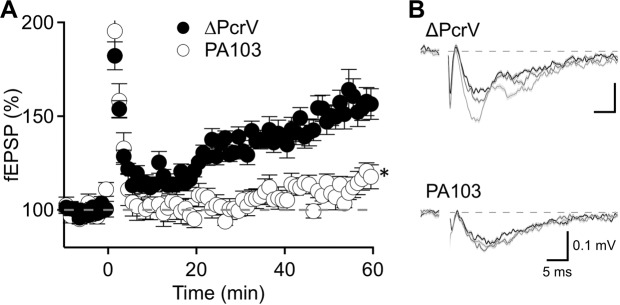
PA103-amyloid in the cerebral ventricle impairs long-term potentiation. Long term potentiation was recorded from evoked field potentials in the hippocampus. (**A**) Plot shows normalized fEPSP slopes, recorded from the hippocampi of ΔPcrV- or PA103-amyloid treated mice, with 10 min baseline, followed by high-frequency stimulation and 60 min recording of response. *p = 0.02; *t*-test comparing fEPSP responses at 55–60 min. (**B**) Representative fEPSP traces of baseline (darkest), at 15 min (medium) and at 60 min (lightest) after stimulation. Each trace depicts averaged 10 fEPSP traces from each time point. Vertical and horizontal bars indicate 0.1 mV and 5 ms, respectively.

Postsynaptic spines on CA1 pyramidal neurons are dynamic; learning and memory increases spine density and enhances spine maturation^24,25^. Thus, we next asked whether the spine density reduction would result in reduced long-term potentiation (LTP), a cellular model for learning and memory. We performed acute brain slice recordings to study LTP at the Schaffer collateral synapses two weeks after animals were ICV injected with ΔPcrV- or PA103-amyloid (3 mice each). Theta burst stimulation-induced synaptic strengthening that lasted for more than 1 hour in ΔPcrV-amyloid brain. However, in PA103-amyloid treated brain, LTP was significantly reduced (ΔPcrV: 158 ± 10.7%; PA103: 115 ± 4.3%; n = 4 slices each, *p* = 0.02, *t*-test; Fig. 4). This finding may be attributable to the reduced apical spine density two weeks after ICV injection, and is consistent with our previous study that showed mice receiving ICV PA103-amyloid have impaired learning and memory^15^. These results, taken together with the findings in dye-dialyzed cells, suggest that PA103-amyloid reduces CA1 spine density and impairs animal learning.

### Route of entry and association with time-dependent spine density reduction

**Figure 5:**
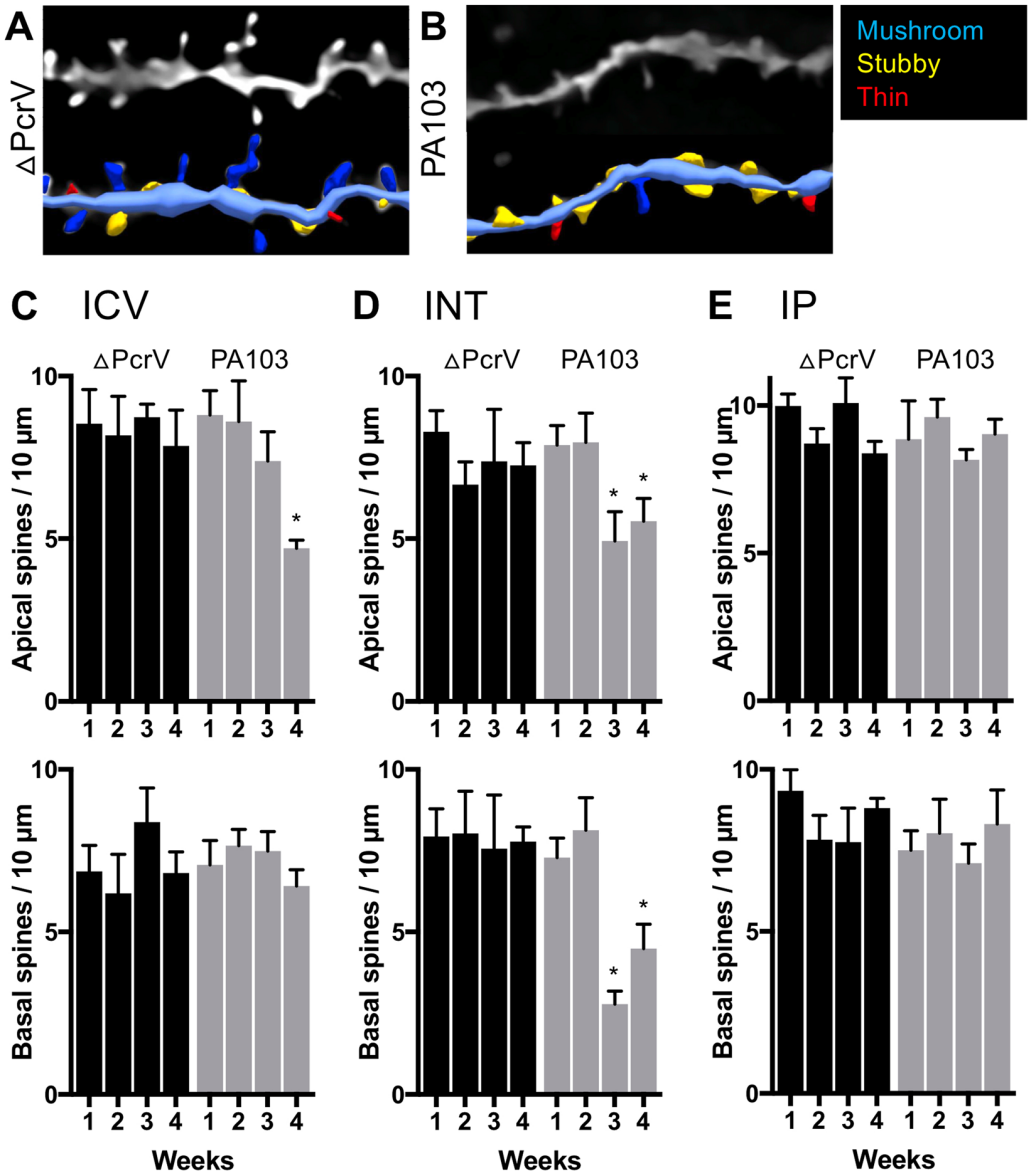
Time-dependent changes of dendritic spines after intracerebroventricular, Intratracheal, or intraperitoneal injection of PA103-amyloid. (**A,B**) Negative photomicrographs of representative apical dendritic spines obtained from mice ICV injected with ΔPcrV- or PA103-amyloid. The corresponding 3D reconstruction of the dendrites and classified (color-coded) dendritic spines are shown below. (**C**–**E**) Bar graphs depict the mean differences in apical (upper) and basal (lower panel) dendritic spine density in mice treated with ΔPcrV- or PA103-amyloid via intracerebroventricular injection, intratracheal instillation, or intraperitoneal injection. *Statistical significance comparing to the first week using ANOVA with Tukey’s *post-hoc* test.

To further quantify the effects of ICV injected PA103-amyloid on dendritic spine reduction over a prolonged time period, we utilized Golgi-impregnation of CA1 pyramidal neurons. Golgi stained neurons were similar in morphology to Lucifer yellow dialyzed cells. We offline reconstructed the distal and proximal (oblique or lateral) apical segment and quantified spine density and type from each dendritic branch (Fig. 5A,B). Consistent with the results from dye-filled neurons, spines on apical dendritic trees were susceptible to PA103-amyloid neurotoxicity. Specifically, the spine density on the apical proximal dendrites was reduced at weeks 3 and 4, whereas that on the apical distal dendrite showed reduction only at week 4. No other overt differences were noted between the apical proximal and distal dendritic branches. The basal dendrites were quantified similarly. Results from distal and proximal segments were similar, and PA103- or ΔPcrV-amyloid did not affect spine density or spine type. Thus, results from proximal and distal dendrites were pooled (Fig. 5C). We report that apical spine density was significantly lower at 4 weeks post ICV PA103-amyloid (4.7 ± 0.3; n = 8 cells per week; *p* = 0.0013; ANOVA with Tukey’s *post-hoc* compared to week 1: 8.8 ± 0.7). In contrast, no significant changes in spine density were noted in basal dendrites.

Our results indicate that lung endothelial-derived cytotoxic amyloids, when present in the cerebrospinal fluid perfusing the brain, reduce apical dendritic spine density in CA1 pyramidal neurons. Importantly, non-cytotoxic lung-derived amyloids do not impair brain neurons. Because ΔPcrV- and PA103-amyloid are lung-derived amyloids, and because previous studies have demonstrated that amyloids extracted from neuropathologic diseased brains translocate to the brain when injected into the systemic circulation^16–18^, we next examined whether intratracheal (INT) instilled or intraperitoneal (IP) injected endothelial amyloids would reduce CA1 dendritic spine density. Similar Golgi stain quantification was carried out as described following the ICV injection. Results showed that PA103-amyloid INT not only reduced apical spine density earlier (Week 3: 4.9 ± 0.9; *p* = 0.010; Week 4: 5.5 ± 0.7; *p* = 0.026; n = 8 cells per week, ANOVA with Tukey’s *post-hoc* compared to week 1: 7.9 ± 0.6; Fig. 5D), but also reduced basal spine density at 3 and 4 weeks post airway instillation (Week 3: 2.8 ± 0.4; *p* = 0.001; Week 4: 4.5 ± 0.7; *p* = 0.003; n = 8 cells per week, ANOVA with Tukey’s *post hoc* compared to week 1: 7.3 ± 0.6). However, endothelial amyloids delivered IP did not affect spine density (Fig. 5E).

### Endothelial amyloids modulate spine types in apical and basal dendrites differently

**Figure 6:**
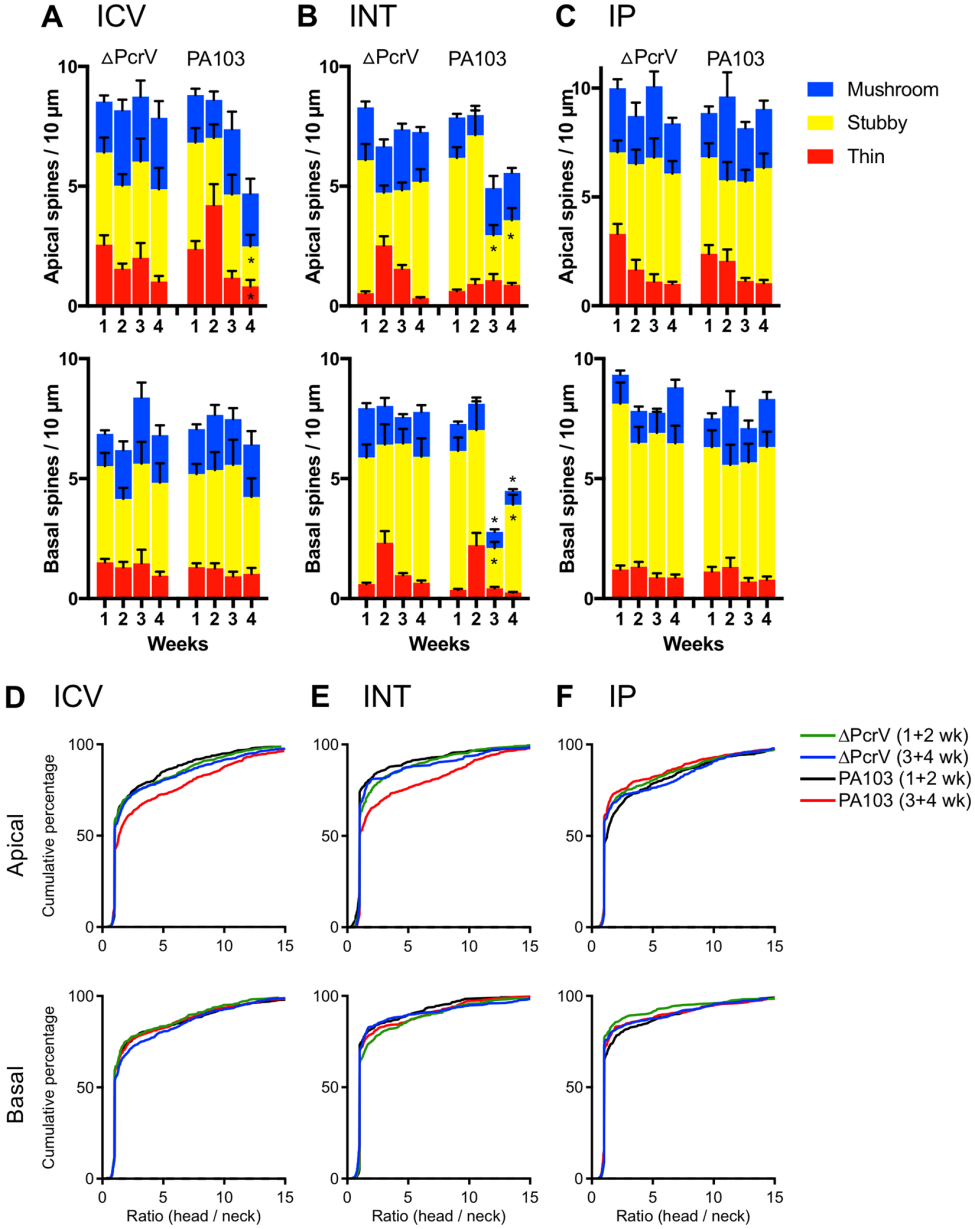
Time-dependent changes in dendritic spine type distribution via PA103-amyloid injection routes. (**A**–**C**) Bar graphs summarize the spine types in apical (upper) and basal (lower panel) dendrites in mice treated with ΔPcrV- or PA103-amyloid via intracerebroventricular injection, intratracheal instillation, or intraperitoneal injection. Statistical comparisons were made in the treatments that showed spine density reduction. *Statistical significance comparing to the first week using *t*-test. (**D**–**F**) Cumulative frequency (%) of the shape parameter (spine head diameter/neck diameter) of apical and basal dendrites. Color traces show spine shape parameter calculated from weeks 1 and 2, or weeks 3 and 4 in mice treated with ΔPcrV- or PA103-amyloid.

Next, spines were further classified by their anatomical shape (i.e., mushroom, stubby, or thin), and we plotted the spine-type density results as bar graphs (Fig. 6). Results showed that the reduced spine density 4 weeks post ICV PA103-amyloid injection was attributed to decreased stubby and thin spines (Week 4: stubby: 1.7 ± 0.5, *p* = 0.015; thin: 0.82 ± 0.27, *p* = 0.002; n = 8; *t*-tests comparing vs. week 1: stubby: 4.4 ± 0.6; thin: 2.4 ± 0.3; Fig. 6A). After INT PA103-amyloid instillation, the decline in apical spine density at 3 and 4 weeks was only attributable to reduced stubby spines (Week 3: 1.9 ± 0.4, *p* = 0.016; Week 4: 2.7 ± 0.5, *p* = 0.021; n = 8; *t*-tests compared with week 1: 5.6 ± 0.4; Fig. 6B upper panel). However, in basal dendrites the decrease in spine density was due to reduced mushroom and stubby spines (Week 3: mushroom: 0.68 ± 0.11, *p* = 0.042; stubby: 1.7 ± 0.3, *p* = 0.003; Week 4: mushroom: 0.58 ± 0.07, *p* = 0.039; stubby: 3.7 ± 0.4, *p* = 0.028; n = 8; *t*-tests compared with week 1: mushroom: 1.1 ± 0.1; stubby: 5.8 ± 0.6; Fig. 6B lower panel).

We further quantified changes in spine shape by calculating the ratios of spine head-to-neck diameters, because this calculation would most sensitively reflect a shift between mushroom and stubby/thin spines. A mushroom spine would have a ratio >1.1, whereas the ratio would be ≤1.1 for stubby or thin spines, as classified by our parameters (see Methods for detail). The ratios from weeks 1 and 2, and those from weeks 3 and 4 after treatment were expressed as cumulative frequency traces (Fig. 6D–F). Our results showed that after ICV- and INT-injection of PA103-amyloid, the curves for apical dendritic spines were right shifted (i.e., head/neck diameter ratio increased), suggesting either an increase in mushroom or a reduction in stubby/thin spines (Fig. 6D,E, upper panel). Interestingly, although the basal dendritic spine density was reduced after INT PA103-amyloid injection, the cumulative frequency trace was unaffected, suggesting that simultaneous reductions in both mushroom and stubby spines likely cancel each other, resulting in unchanged head/neck diameter ratio cumulative frequency (Fig. 6E, lower panel).

Overall, the results showed that whereas the mushroom spines maintained their anatomical structure in apical dendrites after PA103-amyloid was introduced into the ICV or INT route, they were reduced in basal dendrites after INT-instillation. In toto, there was a disproportionate reduction in apical dendritic spine types, whereas the spine type distribution was maintained in basal dendrites, despite an overall reduction in the spine density. Thus, PA103-induced endothelial cell cytotoxic amyloids: (i) cause dendritic spine retraction, (ii) reduce both apical and basal dendritic spine density when delivered through the airway, (iii) impair thin and stubby spines in apical dendrites, and iv) reduce mushroom and stubby spines and reduce basal spine density when introduced through the airway. Our work is consistent with a working model where cytotoxic endothelial amyloids are transmissible in the lung; amyloids that enter the brain cause dendritic spine pruning and progressive neuropathology.

## Discussion

Nosocomial pneumonia induces the production of cytotoxic amyloids from pulmonary endothelium^8,14^. These amyloids can be recovered from bronchoalveolar lavage and cerebrospinal fluids of patients harboring monomicrobial *P. aeruginosa, Klebsiella pneumoniae*, and *Staphylococcus aureus* lung infections. Generation of these cytotoxic species seems to require the presence of bacterial virulence factors. For example, the production of cytotoxic endothelial amyloids is dependent upon the *P. aeruginosa* type 3 secretion system and its exoenzymes; i.e., *P. aeruginosa* strains and mutants lacking the type 3 secretion system and its effectors do not generate cytotoxic amyloids^8,15^. Delivering the endothelial-derived cytotoxic amyloids directly into the cerebral ventricle impairs animal learning and memory^15^. Here, we show that the infection-elicited lung endothelial amyloids reduce neuronal dendritic spine density, and further, that amyloid delivery into the airway is sufficient to cause this evolving neurotoxicity. Because these amyloids propagate and are transmissible among endothelial cells^13,14^, our findings further suggest that when reintroduced into a naïve lung, the amyloid load may amplify or be sustainable over time. Thus, once lung bacterial infection elicits cytotoxic amyloid production, the amyloid burden may be prevalent even in the absence of an active infection. While the initiating cause of cytotoxic amyloid production is lung infection, we show that both the primary infection and introduction of the cytotoxic amyloid directly into the lung, in the absence of infection, results in an insidious neuropathy characterized by impaired long term potentiation and decreased dendritic spine density.

Approaches to visualize and quantify dendritic spines have significantly advanced since the pioneering work of Santiago Ramón y Cajal (see review^26^). In this study, we utilized fluorescence dye labeling with high resolution optical imaging and Golgi impregnation methods, both of which have been used extensively to quantify changes in postsynaptic spines, with results comparable to electron microscopy quantifications^27–29^. Comparing between the two methods, our results indicate that dialysis labeling was more sensitive to detect spine density change because following ICV injection, fluorescence labeled cells showed reduction at two weeks, while the reduction in Golgi impregnated cells became significant after one month. Further, shifts in spine types differ between the two methods after PA103-amyloid ICV injection. Although we did not further explore the difference, both methods showed that only apical spines were reduced after ICV injection. The postsynaptic spines on a pyramidal neuron are an essential component of the excitatory synapses. Disease-associated or amyloid-induced dendritic pruning may involve neurotoxicity and neuroinflammatory responses, including the activation of the complement, astrocytes, and microglia^30–32^. The mechanism involved in spine density reduction and spine remodeling induced by lung-derived amyloids is currently unknown and beyond the scope of the current study; it is an active focus of our ongoing work. Nevertheless, our ongoing preliminary results show that the prevalence of activated microglia and astrogliosis is increased in animals receiving the detrimental endothelial amyloids (data not shown), suggesting neuroinflammation is likely involved.

The clearance of brain amyloids from the cerebrospinal fluid into the blood is a physiological process that is needed to maintain healthy brain function^33^. Conversely, exogenous amyloid species that are extracted from diseased brains and injected into either the blood or peritoneum are capable of circulating through the blood and accessing the central nervous system, where they accumulate and propagate. Notably, these diseased amyloid species seed in the same brain region from which they originated, damaging the same type of neurons most susceptible to the amyloid species, and recreating neurologic deficits^16,18,19,34^. A key aspect of the current study is that the cytotoxic endothelial amyloid species studied were generated from cells that were isolated from the lung. When administered back into the lung, the amyloids may amplify in the lung or elsewhere, or increase their cytotoxic potential over time, and disseminate into the brain where they cause dendritic pruning. The neurotoxicity of endothelial amyloids is dependent upon the *P. aeruginosa* type 3 secretion system and its virulence factors, because ΔPcrV-infected cells do not produce amyloids that impair animal learning, long-term potentiation, or dendritic spine density. It is notable that these endothelial-derived cytotoxic amyloids act like prions, are immunoreactive to Aβ and τ antibodies, and are generated in the lung during lung infection, yet they possess neurotropic properties.

Although we did not quantify the translocation of endothelial amyloids from the peritoneum to the blood in this study, IP injected PA103-amyloid (300 µL) did not reduce spine density. These results suggest that either too little injected amyloids were absorbed into the circulation, or that this route of entry would require a longer time to translocate and damage neurons in the brain^16,35^. The injected amyloids are bacterium-free; the sepsis-elicited upregulation of the receptor for advanced glycation end products (RAGE) that transports humoral amyloids into the brain parenchyma should be absent^36^. Notably, and in stark contrast, airway instillation of PA103-amyloid significantly reduced spine density, suggesting that endothelial amyloids applied into the airway propagate in a way that exacerbates damage to neurons, even compared to ICV injection.

ICV injected PA103-amyloids reduced the dendritic spine density, but this effect was restricted to the apical dendrites within the stratum radiatum and lacunosum moleculare layers. On the other hand, INT injected amyloids impaired both apical and basal dendrites—essentially all dendritic inputs to a CA1 pyramidal cell. Interestingly, the reductions in apical and basal dendritic spines were differentially affected. Based upon the classification of spine types, our results showed that stubby and thin spines were reduced after ICV and INT injection, whereas mushroom spines remained intact in apical dendrites. To further quantify this change, we sorted the spines by the head-to-neck diameter ratios because this calculation would distinguish mushroom spines (that have large head/neck diameter ratios) from stubby or thin spines (small ratios). Cumulative frequency plots showed that ICV and INT injection increased the spine head/neck diameter ratios, consistent with a reduction in stubby or thin spine density. In contrast with apical dendritic spines, the reduction in basal dendritic spine density was due to both decreased mushroom and stubby spines after INT injection. The opposite effects of mushroom and stubby on head/neck diameter ratios effectively canceled each other, resulting in an unchanged cumulative frequency. While this finding was unexpected, CA1 pyramidal neurons receive inputs from CA3 neurons (i.e., Schaffer collateral pathway) and the entorhinal cortex (i.e., performant pathway). It is possible that INT applied amyloids are capable of accessing the brain via retrograde nasal^37–39^ or nerve innervation^40^ that specifically affects the basal dendritic inputs of CA1 cells. In the light of this, studies have implicated that the nasal/olfactory connection provides a direct route to the brain for therapeutic interventions^41^. We think this is an unlikely mechanism to account for our findings because amyloids were instilled into the trachea, below the vocal cords, and mice neither sneeze nor regurgitate, even if applied amyloids are swallowed. Nevertheless, it is evident that amyloids, and thus its associated neuropathology, spread via neuron connectivity, making the nerves that innervate the lung a plausible retrograde amyloid route^42^.

Currently, the underlying mechanism(s) to explain why amyloids introduced into the airway lead to more severe neural damage is unclear, although we offer the following considerations: (1) The amount of INT applied amyloids (50 µL) was simply higher than that injected ICV (1.5 µL); and (2) INT applied amyloids may further amplify or increase in potency in either the lung or the brain. To the first point, we used 50 µL INT because we routinely instill 40–50 µL of live bacteria into the airway to ensure that enough solution is distributed throughout alveoli. Nevertheless, the volume of INT injected amyloids was 33-fold higher than the ICV injected amyloids. The second point is especially important. For the instilled amyloids to propagate in lung endothelium^14^, they need to cross or cause injury to the epithelial layer. Alternatively, it is also possible that the amyloids are capable of propagating in lung epithelium or other cell types. Pulmonary alveoli occupy a large surface area, and each alveolus is supplemented with multiple capillaries necessary for efficient blood gas exchange. It is likely that exposing epithelium and endothelium to lung-derived amyloids elicits further production of lung-derived amyloids, which can be released into the circulation, distributed through the blood to the brain, resulting in dendritic spine pruning.

In a critical care setting where bacterial pneumonia patients receive antibiotics to clear the virulent bacteria organisms, cytotoxic amyloids can linger in the airway, lung, or circulation, and they may initiate a neuropathology cascade not previously recognized. This study provides the first direct evidence that lung-derived amyloids impair neuronal anatomy, with dendritic spine pruning.

## Methods

### Preparation of pulmonary endothelial amyloids

Endothelial amyloids were prepared and collected from cell supernatant using previously described methods^13,14^. Briefly, both *P. aeruginosa* strain PA103 and the non-virulent control strain ΔPcrV were used. Prior to infection, bacterial strains were streaked from frozen stocks onto Vogel-Bonner minimal salts media and incubated overnight at 37 °C. Bacteria were collected and diluted in sterile phosphate-buffered saline (PBS) to the colony forming unit/mL (CFU/mL) ratio of 2 × 10^8^ CFU/mL. Confluent monolayers of primary pulmonary microvascular endothelial cells obtained from Sprague-Dawley lungs were inoculated with either PA103 or ΔPcrV at a multiplicity of infection (MOI) of 20:1. The supernatant was collected 4 hours after inoculation, centrifuged for 10 min at 2,000 × g, and sterilized by passage through a 0.22 µm filter to ensure that endothelial supernatants containing amyloid (i.e., PA103-amyloid and ΔPcrV-amyloid) were free of bacteria, which was confirmed subsequently with negative plating studies. These endothelial supernatants contained either cytotoxic or non-cytotoxic amyloids, dependent upon whether the bacterial strain possessed a function type 3 secretion system^14,43,44^. Whereas the endothelial supernatant obtained post ΔPcrV infection (i.e., ΔPcrV-amyloid) is not cytotoxic to endothelium and does not cause gap formation in endothelial monolayers, the supernatant obtained post PA103 infection (i.e., PA103-amyloid) is cytotoxic, disrupts endothelial monolayers *in vitro*, and impairs learning *in vivo*. Notably, the cytotoxicity in PA103-amyloid is attributable to beta-amyloid (Aβ) and tau (τ) oligomers, as demonstrated in our previous studies^44^. Removal of these endothelial amyloid proteins (Aβ and τ) using immunoprecipitation removes the cytotoxicity, whereas amyloid proteins eluted from antibody complexes rescue cytotoxicity. Thus, herein endothelial supernatants obtained post PA103- or ΔPcrV-infection is denoted as PA103-amyloid or ΔPcrV-amyloid, respectively.

### Mice

Subjects were 13- to 17-week old C57BL/6 J male mice. All procedures were conducted in accordance with the guidelines described in the National Institutes of Health Guide for the Care and Use of Laboratory Animals. The University of South Alabama Institutional Animal Care and Use Committee approved all procedures. The following injection procedures were performed on mice after they reached an anesthesia plane with ketamine/xylazine (90/5 mg/kg, intraperitoneal).

Intracerebroventricular (ICV) injection was performed using a stereotaxic method similar to our previous description^15,45^. Briefly, mice were mounted on a stereotaxic platform with a motorized injector (UMP3T-1; World Precision Instruments, Sarasota, FL). A bolus of 1.5 µL endothelial-derived amyloid (i.e. PA103-amyloid or ΔPcrV-amyloid) was delivered at 0.2 µL/min into the lateral ventricles of both hemispheres. After each injection, we allowed the needle to rest for 5 min prior to withdrawal. Intraperitoneal (IP) injection was performed with a bolus of 300 µL PA103-amyloid or ΔPcrV-amyloid. Intratracheal (INT) instillation was performed similar to our animal infection methods described previously^15^, except that endothelial-derived amyloids were used. Briefly, a bolus of 50 µL PA103-amyloid or ΔPcrV-amyloid was delivered into the airway of a mouse. After each injection, we allowed the animal to stay on a 35° surgical station for 1–2 min prior to wound closure. Animals that recovered from the procedures were euthanized and brains were collected at specified time points.

### Brain slice preparation and recording

A total of six mice (3 each for PA103- and ΔPcrV-amyloid ICV injection) were used for brain slice recording and intracellular dye-filling studies. Two weeks after ICV, deeply anesthetized mice were euthanized by decapitation, acute hippocampal slices were prepared^8^, and recording was performed^46–48^ with the following modifications. Theta burst stimulation was delivered >10 min following a stable baseline. Theta burst stimulation consisted of a single burst of 5 stimulations at 100 Hz, 10 bursts delivered at 5 Hz per sweep, and 3 sweeps delivered at 10 s interval. Recordings were obtained using an EPC10 amplifier (HEKA). Analog signals were further amplified 10x and filtered at 5 kHz using an Axopatch amplifier (Axon Instrument) and digitized at 20 kHz using Patchmaster software (HEKA). For histology, fixed and Golgi stained brains were sliced at 70 µm in 30% sucrose PBS.

### Intracellular dye-filling and imaging

Pyramidal neurons in the region CA1 were visualized with a Leica DMLFS microscope with Nomarski DIC/IR and a modified Dodt gradient contrast optics, equipped with an Andor Revolution DSD2 unit. Using a whole-cell patch-clamp configuration, we dialyzed the cells for 10 min with an internal solution containing (in mM) 140 KMeSO_4_, 8 NaCl, 1 MgCl_2_, 10 HEPES, 5 MgATP, 0.05 EGTA, and 1 Lucifer yellow CH (pH 7.3)^49^. We monitored access resistance, input resistance, and membrane potential every 3 min during the dialysis. Neurons were included only if they had a resting potential between −70 to −55 mV, a stable input resistance, and overshooting action potentials. The input and series resistance were determined from a ~30 pA (500 ms) hyperpolarizing current injection pulse. At completion, the pipette was withdrawn gently, and the brain slice was fixed in 4% paraformaldehyde for 1 hr at room temperature. Fixed brain slices were washed 3 times, 10 min each in 0.1 M PBS before imaging on a Nikon A1-R laser confocal microscope.

### Golgi staining and histology

Golgi-Cox staining was prepared using the commercially available FD Rapid GolgiStain Kit (FD NeuroTechnologies, Columba, MD), by following the provided procedures. Briefly, after animals were deeply anesthetized, they were transcardially perfused with saline. Brains were obtained, rinsed in double distilled water, and immersed in the Golgi-impregnation solution. The impregnation solution was replaced after 24 hr and then kept in the dark for 2 weeks. After Golgi-impregnation, brains were transferred to a clearing solution for 3 days, with one solution replacement in the first 24 hr. Brain slices prepared at 70 µm were mounted on gelatin-coated glass slides (FD NeuroTechnologies), incubated in silver nitrate, rinsed in distilled water, dehydrated in graded ethanols, cleared in xylene, and coverslipped under Permount, as instructed in the kit. Images were captured with a 60x oil lens mounted on a Nikon Eclipse 80i microscope.

### Spine quantification and 3D remodeling

Captured microscopic images from single-cell dialysis and Golgi staining preparations were similarly processed (Fig. 1). Briefly, raw images from the same setting were compiled into a z-stack of TIFF format images using ImageJ based FIJI (NIH^50^), deconvoluted using the algorithms tailored to each microscope system and lens in Huygens suite (Scientific Volume Imaging B.V., Netherlands), and manually traced using Neurolucida (MBF Bioscience, Williston, VT). The analysis of spines was performed in CA1 pyramidal neuronal segments visible for at least 30 µm. Automated dendritic spines detection was performed using the following setting: outer range 3.0 µm, minimum height 0.5 µm, detection sensitivity 90%, and minimum voxel count 30. Spine classification was performed using the following setting: head-to-neck diameter ratio 1.1, length-to-head ratio 2.5, mushroom head size 0.35 µm, and filopodium length 3 µm. To assess changes in spine morphology, spines in the selected segments were classified into mushroom, stubby, thin, and filopodia spines using the predefined criteria, and the proportion of spines in each category was calculated for each neuron. Dendritic spine density was expressed as the number of spines per 10 µm dendritic length. The relative distribution of spine type was calculated from each type of spines normalized to the total number of spines and expressed as a percentage.

### Experimental design, data and statistical analyses

To test the hypothesis that lung endothelial-derived amyloids within the cerebrospinal fluid damage neuronal spine morphology, endothelial-derived amyloids were ICV injected into the cerebral lateral ventricle of mice. To further compare whether endothelial-derived amyloids in the lung or gut/systemic circulation would cause similar damages to neurons, INT instillation or IP injections were performed, respectively. Lucifer yellow-filled neurons were pre-selected during recording and dialysis. After the compilation of images, CA1 pyramidal neurons were determined to be well-filled with Lucifer Yellow if a single cell body and evenly stained dendrites could be followed. Stained neurons that showed patchy or interrupted dendrites were excluded; however, dendritic spines were not used as a criterion for the staining quality. For each cell, dendritic spine density was measured on four dendritic segments in the apical dendritic tree within a range of 150–300 µm from the cell body.

Golgi impregnated cells were quantified similarly to dye-filled cells with the following differences: The density of dendritic spines was quantified on proximal (oblique or lateral; 150–300 µm from somata) and distal (within the stratum lacunosum-moleculare layer; >400 µm from somata) branches of the apical dendritic trees. Dendritic spine density was also quantified from proximal (~50 µm) and distal (>100 µm) segments of the basal dendrites (in stratum oriens). Four mice per treatment per week were performed (n = 4 mice). Four cells per dendritic branch (i.e., apical proximal, apical distal, basal proximal, or basal distal) were quantified, and results from the same dendritic branch were averaged for each animal. Spine density numbers of apical proximal and apical distal from each animal were used for statistical analyses and plots (i.e., n = 2 per mouse). This also applies to basal apical/distal dendritic analyses and plots. Statistical analyses were calculated was we previously described^15^. Briefly, offline data analysis was tabulated using custom macros written in Igor Pro (WaveMatrics) and Excel (Microsoft); statistical comparisons were performed with Igor Pro or Prism (Graphpad). Data are expressed as mean ± s.e.m., and were subjected to *t*-test or analysis of variance (ANOVA) followed by *post-hoc* Tukey’s comparison (as specified).

## Data Availability

All data generated and/or analyzed during the current study are available from the corresponding author on reasonable request.
